# Antimicrobial Efficacy of Silver Diamine Fluoride against *Enterococcus faecalis*: A Systematic Review of *In Vitro* Studies

**DOI:** 10.1155/2022/6544292

**Published:** 2022-12-16

**Authors:** Juzer Shabbir, Zohaib Khurshid, Muhammad Sohail Zafar, Waqas Ahmed Farooqui, Eisha Imran, Shariq Najeeb, Sompop Bencharit

**Affiliations:** ^1^Department of Operative Dentistry and Endodontics, Baqai Medical University, Karachi, Pakistan; ^2^Department of Prosthodontics and Dental Implantology, College of Dentistry, King Faisal University, Al Ahsa, Saudi Arabia; ^3^Center of Excellence for Regenerative Dentistry, Department of Anatomy, Faculty of Dentistry, Chulalongkorn University, Bangkok 10330, Thailand; ^4^Department of Restorative Dentistry, College of Dentistry, Taibah University, Al Madinah, Al Munawwarah 41311, Saudi Arabia; ^5^Department of Dental Materials, Islamic International Dental College, Riphah International University, Islamabad 44000, Pakistan; ^6^School of Public Health, Dow University of Health Sciences, Karachi, Pakistan; ^7^Department of Dental Materials, Islamabad Medical and Dental College (IMDC), Islamabad, Pakistan; ^8^Schulich School of Medicine and Dentistry, Western University, London, Ontario, Canada; ^9^Evidentia Dental Reseach, Calgary, Alberta, Canada; ^10^Department of Oral and Molecular Craniofacial Biology, Philips Institute for Oral Health Research, School of Dentistry, Virginia Commonwealth University, Richmond, VA 23298, USA; ^11^Department of Biomedical Engineering, College of Engineering, Virginia Commonwealth University, Richmond, VA 23220, USA; ^12^Department of Oral Rehabilitation, The James B. Edwards College of Dental Medicine, Medical University of South Carolina, Charleston, SC 29425, USA

## Abstract

*Introduction/Objectives*. *Enterococcus faecalis* has been implicated in infections of treated root canals. Current irrigants and intracanal medicaments cannot eliminate *E. faecalis* completely from the root canal. Silver diamine fluoride (SDF) prevents caries by promoting remineralization and exerting an antibacterial effect. Studies suggest that SDF may possess antibacterial properties against *E. faecalis*. The purpose of this review is to systematically and critically analyze the literature, focusing on the use of SDF as an intracanal medicament or irrigant, when compared to other antibacterial agents. *Data/Sources*. The focused question was “Is the antibacterial effect of SDF against *E. faecalis* better than other intracanal medicaments and irrigants?” Using the keywords ((silver diamine fluoride) AND (*Enterococcus faecalis*)) AND ((sodium hypochlorite) OR (NaOCl) OR (chlorhexidine) OR (calcium hydroxide) OR (Ca(OH)_2_)), an electronic search was conducted on the following databases: PubMed/MEDLINE, ISI Web of Science, Scopus, EMBASE, and Google Scholar. The clinical trial registers ClinicalTrials.gov and CONTROL were also searched using the same keywords. General characteristics and outcomes were extracted, and quality of the studies was assessed using the Preferred Reporting Items for Laboratory studies in Endodontology (PRILE) guidelines. *Study Selection*. Six articles (five *in vitro* studies and one *ex vivo* study) were included in this systematic review. In the majority of the studies, SDF had equal or better antibacterial efficacy against *E. faecalis* compared to calcium hydroxide, sodium hypochlorite, and chlorhexidine. However, the majority of the studies did not fulfill several items in the PRILE criteria and had numerous sources of bias. *Conclusions*. Within the limitations of the systematic review and the studies reviewed, SDF may be effective against *E. faecalis* and therefore could be used as an intracanal medicament and/or irrigant to prevent reinfections of the root canals and improve the outcomes of endodontic treatment. However, animal and clinical studies should be carried out to determine the efficacy of SDF in endodontics. *Trial Registration*. The protocol for this review was registered on PROSPERO. Registration number: CRD42021224741.

## 1. Introduction

*Enterococcus faecalis* is a facultative anaerobic Gram-positive bacterium associated with endodontic infections and periapical periodontitis [1, 2] and implicated in reinfection of endodontically treated root canals [1]. Removal of infected or inflamed dental pulp and dentine, through mechanical and chemical measures, is an important part of routine endodontic therapy to reduce recurrent or reinfection of the root canal [3, 4]. Sodium hypochlorite (NaOCl) [5]\chlorhexidine and calcium hydroxide (Ca(OH)_2_) [6] have been used as intracanal medicaments to minimize the bacterial growth within the root canal. However, these chemicals have a number of disadvantages. NaOCl can cause severe adverse reactions, such as tissue damage, pain, and hematoma, if the irrigant is accidentally injected beyond the root apex [7]. Ca(OH)_2_, a common intracanal medicament and sealer, can suppress microbial growth in the root canals through its alkaline pH [8, 9]. Chlorhexidine is also an intracanal medicament used as disinfectant [10]. *E. faecalis* has been shown to survive chlorhexidine and Ca(OH)_2_ [11]. The inefficiency of *E. faecalis* elimination by NaOCl, Ca(OH)_2_, or chlorhexidine renders the search for an alternative solution necessary.

Silver diamine fluoride (SDF) is a colorless liquid used to prevent and arrest dental caries, as well as reduce dentine sensitivity [12]. SDF is usually applied as a professional varnish as a prophylactic measure against dental caries [13]. SDF prevents and arrests caries due to the presence of silver and fluoride. At a pH of 10.4, SDF contains 24.4% to 28% silver and 5.0 to 5.9% fluoride [14]. Silver exerts an antimicrobial effect while fluoride promotes remineralization [15]. Silver ions inhibit DNA replication, denature proteins, and disrupt cell membranes to impede bacterial growth [16]. SDF also exerts its antibacterial effect by forming organometallic complexes inside the bacteria [17], which can induce bacterial cell wall rupture, block bacterial enzymatic function, and interfere with DNA replication. SDF has been studied as a potential intracanal irrigant and medicament to disinfect the root canal [18], in particular its effects against *E. faecalis* [19]. Clinical and laboratory evidence for the effectiveness of SDF against *E. faecalis* associated with endodontic reinfection is not clear. This systematic review therefore is aimed at summarizing the current literature on the effectiveness of SDF against *E. faecalis* associated with endodontic reinfection.

## 2. Materials and Methods

### 2.1. Focused Question

The protocol for this review was registered on PROSPERO (registration number CRD42021224741). Using the Participants, Intervention, Control, and Outcomes (PICO) principal stated in the Preferred Reporting Items for Systematic Reviews and Meta-Analyses (PRISMA) guidelines [20, 21], a focused question was constructed (Supplemental Information 1). The focused question was “Is the antibacterial effect of SDF against *E. faecalis* better than other intracanal medicaments and irrigants?”

### 2.2. Eligibility Criteria

Before conducting the literature search, eligibility criteria were formulated. The following types of articles were included:

1. Studies comparing antibacterial efficacy of SDF against *E. faecalis* with other intracanal irrigants and medicaments
2. Clinical studies
3. Animal studies
4. Laboratory studies

The following types of articles were excluded:

1. Reviews
2. Letters to the editor
3. Commentaries
4. Studies not in English

### 2.3. Literature Search

**Figure 1:**
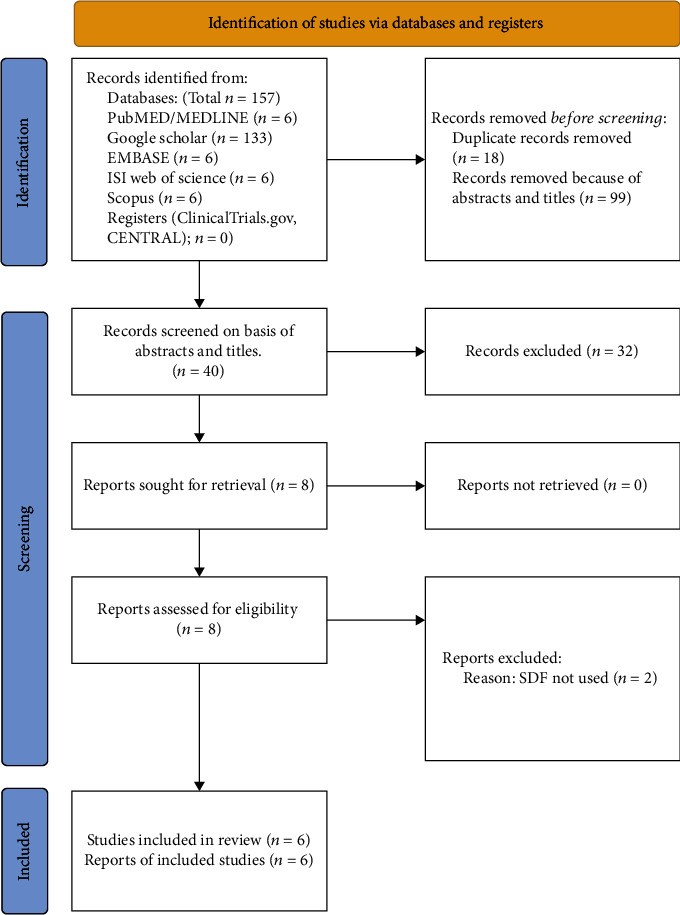
A PRISMA (Preferred Reporting Items for Systematic Reviews and Meta-Analyses) flow diagram of the literature conducted in this systematic review. SDF: silver diamine fluoride.

Using the keywords ((silver diamine fluoride) AND (*Enterococcus faecalis*)) AND ((sodium hypochlorite) OR (NaOCl) OR (chlorhexidine) OR (calcium hydroxide) OR (Ca(OH)_2_)), an electronic search was conducted on the following databases: PubMed/MEDLINE, ISI Web of Science, Scopus, EMBASE, and Google Scholar for all studies published from January 2000 to June 2021. The clinical trial registers ClinicalTrials.gov and CONTROL were also searched using the same keywords. The search was conducted by two investigators independently. Any disagreements were solved by discussion. After the primary search, any nonrelevant articles were excluded based on titles and abstracts. Full texts of articles meeting our inclusion criteria were read to verify eligibility. An additional search was conducted by reading the reference lists of the full-text articles included in the primary search. The PRISMA flow diagram depicting the literature search is illustrated in Figure 1.

### 2.4. Data Extraction

A table was designed and constructed by two investigators independently. Studies were categorized and tabulated according to the type of growth medium or samples, study groups, type of microbial testing, exposure time of the antibacterial substances, and the outcomes of the antibacterial testing. A third person investigated, reviewed, and verified the completed table.

### 2.5. Quality Assessment of Studies

The quality of each study was assessed using a modified version of the Preferred Reporting Items for Laboratory studies in Endodontology (PRILE) guidelines [22]. Briefly, various aspects of the study title, abstract, introduction, materials and methods, results, discussion, conclusion, funding and support, and conflicts of interest were taken into consideration to allocate a score to each study. Images were not assessed for quality because the research questions focused on the bacterial cultures. The quality assessment was carried out by two investigators independently. Each study received a score out of 32 according to the number of PRILE criteria fulfilled. Additionally, if a study fulfilled a criterion partially, it was assigned a score of 0.5 in that category. Any disagreements were solved by discussion.

## 3. Results

### 3.1. Literature Search

Primary search resulted in 157 items. After excluding 117 irrelevant articles and removal of duplicates, 40 articles were screened for eligibility on the basis of titles and abstracts. After removal of 32 further articles, full text and reference lists of eight articles were read comprehensively to determine their eligibility. Two articles were excluded because they had not tested the antibacterial effect of SDF [23, 24]. Hence, six articles were included in this systematic review [18, 19, 25–28]. The interexaminer reliability (Kappa) score was calculated as 0.79. The PRISMA flow diagram depicting the literature search methodology employed for this review is illustrated in Figure 1 (See also Tables 1 and 2).

### 3.2. General Characteristics of the Studies

All six studies included in this review were laboratory studies [18, 19, 25–28]. Only three studies mentioned the number of samples included, which ranged from 40 to 70 [18, 25, 28]. In the study by Hiraishi et al., the antibacterial efficacy of 3.8% SDF was compared to that of Ca(OH)_2_, 2% chlorhexidine, 5.25% NaOCl, and 0.9% sodium chloride against *E. faecalis* cultured on membranes (NaCl) [27]. In Mathew et al., the antibacterial efficacy of 3.8% SDF was compared with that of 2% chlorhexidine and 0.9% NaCl in extracted single-rooted teeth [28]. On the other hand, in the study by Al-Madi et al., the antibacterial efficacy of 3.8% SDF was instead compared with that of 2% chlorhexidine, 5.25% NaOCl, and saline against *E. faecalis* on dentine discs [25]. In the study by Abrar et al., 3.8% SDF was compared with antimicrobial photodynamic therapy (aPDT), 2% chlorhexidine, and 5.25% NaOCl (with 17% Ethylenediaminetetraacetic acid) in extracted premolars [25]. Minavi et al. investigated the antibacterial efficacy of 0.38%, 3.8%, and 38% SDF against *E. faecalis* and the substantivity of 3.8% SDF against the bacterium when compared to 5.25% NaOCl, 2% chlorhexidine, and PBS on bovine dentine discs [19]. Briseño-Marroquín et al. compared various concentrations of SDF (3.8%, 9.5%, 19%, and 38%) with and without potassium iodide against bacterial cultures of *E. faecalis* [26]. Colony-forming units (CFUs) were counted before and after treatment to estimate the efficacy of each chemical against the bacterial culture in three studies [26–28]. In two studies, confocal laser scanning microscopy (CLSM) was used [18, 25]. In one study, disc diffusion assays were used to assess the antibacterial efficacy, and scanning electron microscopy (SEM) was used to compare the substantivity of the different antibacterial treatments [19]. Treatment times ranged from 30 seconds to 24 hours [18, 19, 25–28]. The general characteristics of the studies are included in Table 1.

### 3.3. General Outcomes

In Hiraishi et al., SDF and NaOCl were both significantly more effective in reducing *E. faecalis* numbers than Ca(OH)_2_, but there was no difference between the efficacy of SDF and NaOCl [27]. Similarly, in Mathew et al., there was no difference between SDF and 2% chlorhexidine [28]. On the other hand, in Al-Madi et al., NaOCl had a higher antibacterial efficacy than either SDF or chlorhexidine [25]. Abrar et al. found no statistically significant difference in antibacterial efficacy between SDF, aPDT, NaOCl with EDTA, and chlorhexidine [18]. Moreover, in Minavi et al., 38% and 3.8% SDF had comparable antibacterial efficacies, but both were more effective than 0.38% [19]. In the same study, the substantivity of SDF was comparable to chlorhexidine but greater than NaOCl [19]. Finally, results from Briseño-Marroquín et al. indicate that the antibacterial efficacy of various concentrations (3.8%, 9.5%, 19%, and 38%) of SDF is equally effective in reducing the number of *E. faecalis*, and addition of potassium iodide does not have any impact on the antibacterial activity of SDF against *E. faecalis* [26]. The general outcomes of the included studies are included in Table 1.

### 3.4. Quality Assessment

The overall quality assessment scores of the studies (Table 3) ranged from 15.5 to 23 out of 32 [18, 19, 25–28]. None of the studies fulfilled all of the criteria described in the PRILE guidelines. Only one study specified the type of the study conducted in the title [28] while all studies mentioned the subject area in their title [18, 19, 25–28]. Only three studies described a rationale in their abstracts [19, 25, 28]. On the other hand, all studies mentioned specific objectives in their abstracts [18, 19, 25–28]. However, although all abstracts mentioned the materials in the methods [18, 19, 25–28], only two studies also described the statistics used in the studies [18, 25]. All abstracts described the results and conclusions appropriately [18, 19, 25–28]. In their introductions, all studies provided an adequate background of the information relevant to the investigation [18, 19, 25–28] while all studies except Briseño-Marroquín et al. [18, 19, 25, 27, 28] provided a hypothesis in the introduction. Only two studies provided an ethics statement [18, 26] and only one study provided a statement regarding the protection of animal and human rights [26]. It should also be noted that in any of the studies where extracted human or animal teeth were used, no information regarding informed consent (in case of human teeth donors) or conditions of the animals (in case of animal teeth donors) was given [18, 19, 25, 28]. Although all studies included controls in their experiments, three studies did not mention negative or positive controls [19, 26, 28]. All studies described the materials, samples, and instruments in adequate detail [18, 19, 25–28]. Similarly, all studies described different categories and groups adequately [18, 19, 25–28]. On the other hand, only three studies made any mention of replicating the experiments on identical samples [18, 19, 25]. Only one study mentioned any details about the sterilization and disinfection of materials and equipment or any aseptic techniques used [18]. Only two studies mentioned randomization [18, 28]. None of the studies described blinding of operators. Five studies described the statistical tests conducted [18, 19, 25–27], but only three of them mentioned the software used to carry out the statistical calculations [18, 25, 27]. Although all studies described the statistical difference and mean reduction in bacterial count or growth for all groups [18, 19, 25–28], none of the studies mentioned the loss (or lack thereof) of samples during the experiments nor did they calculate the effect size of the treatment. In the discussion, all studies provided an adequate review of the literature correlating to the results [18, 19, 25–28], but four studies did not mention if the hypothesis of the studies were rejected or accepted [19, 25, 26, 28]. All studies described their true significance [18, 19, 25–28]. Only two studies described their limitations and strengths in their discussion [18, 19]. On the other hand, two studies did not suggest the implications of their results on future studies [26, 28]. Although all studies contained explicit conclusions [18, 19, 25–28], three studies did not provide any rationale for their conclusions [19, 25, 26]. Only three studies disclose the funding or grant information [18, 25, 26]. Similarly, only three studies provided statements regarding conflicts of interest [19, 25, 26].

## 4. Discussion

Even given the limitations of this systematic review, it is possible to conclude that SDF may be a promising candidate as an intracanal irrigant or medicament due to its antimicrobial efficacy against *E. faecalis* [18, 19, 25–28]. Prior studies have observed similar findings when examining the antibacterial efficacy of SDF against cariogenic organisms such as *Streptococcus mutans*, *Lactobacillus acidophilus*, and *Actinomyces naeslundii* [29]. As stated previously, silver ions have been found to be a principal antibacterial component of SDF [29]. Although systematic reviews have found SDF more effective in preventing dental caries when compared to fluoride varnish [12, 30], to date no systematic review has been published on the antibacterial efficacy of SDF against *E. faecalis*, the principal organism in endodontic infections and reinfections. To the authors' best knowledge, this is the first study that has attempted to systematically analyze the studies in which SDF has been used against *E. faecalis*.

For a substance to function as an endodontic antimicrobial medicament or irrigant it should exert an adequate antimicrobial activity against the pathogenic bacteria [31]. Furthermore, the material should have sufficient substantivity or the prolonged association between a substrate (such as enamel or dentine) and the antibacterial material [32]. Chlorhexidine, an antimicrobial with a high substantivity, has been used as a prophylactic mouthwash and a root canal medicament to maintain the disinfection of debridement and shaping of the canal [18]. However, the extent of the antimicrobial effect of chlorhexidine against *E. faecalis* is debatable [33]. The study by Minavi et al. suggests that SDF has similar substantivity and antibacterial activity to that of 2% chlorhexidine [19, 28]. Interestingly, the study by Al-Madi et al. suggests that SDF may in fact have a higher antimicrobial activity than chlorhexidine, making it a promising candidate for long-term disinfection of root canals [25].

Ca(OH)_2_ is a commonly used root canal sealer that also exerts an antibacterial effect due to its alkaline pH. However, *E. faecalis* is able to survive even in the high pH of Ca(OH)_2_, possibly due to a proton pump survival mechanism associated with the cell wall [1]. Therefore, there have been concerns regarding the survival of *E. faecalis* in treated endodontic canals, which can cause reinfection [8]. Using a material such as SDF may overcome this problem if used in conjunction with Ca(OH)_2_. Nevertheless, no studies to date have attempted to compare the sealing ability and antibacterial efficacy of SDF when used in the presence or absence of Ca(OH)_2_.

NaOCl is effective in dissolving organic material and disinfecting canals, but it has low substantivity when compared to chlorhexidine and SDF [19]. Additionally, NaOCl reduces the efficacy of chlorhexidine by reacting with it and forming a precipitate [34]. Furthermore, NaOCl can cause serious adverse effects if extruded past the apical foramen [35]. The study by Hiraishi et al. suggests that SDF may have an antibacterial efficacy similar to that of NaOCl [27], indicating that SDF may integrate the antibacterial efficacy of NaOCl with the substantivity of chlorhexidine. Nevertheless, another study found the antibacterial efficacy of SDF inferior to that of NaOCl but still greater than chlorhexidine [25]. In other studies, there was no significant difference in the antibacterial efficacy of SDF and other endodontic medicaments [18, 28]. Therefore, more studies are needed before the antibacterial efficacy of SDF can be established.

Although most studies reviewed in this systematic review suggest that SDF has an antibacterial effect against *E. faecalis*, there were several limitations present. Firstly, all of the studies in this review were *in vitro* studies [18, 19, 25–28]. The oral environment *in vivo* has several factors which cannot be readily replicated in a laboratory environment. Masticatory forces, ingress of saliva, and parafunctional habits may impact the outcomes of endodontic therapy. Additionally, the quality assessment of the studies revealed a number of sources of bias and deficiencies in the studies reviewed. Particularly, most studies did not employ operator blinding or sample randomization. In addition, the outcomes of this systematic review have limitations. Finally, due to the heterogeneity and *in vitro* nature of the studies, no meta-analysis could be conducted. Therefore, the overall treatment effect of SDF cannot yet be estimated. Therefore, to ascertain the clinical efficacy of SDF in preventing endodontic infections and reinfections, more studies are needed. In particular, animal and human trials should be conducted to assess the feasibility of SDF as an intracanal medicament.

## 5. Conclusion

Within the limitations of the systematic review and the studies reviewed, SDF may be effective against *E. faecalis* and therefore be used as an intracanal medicament and/or irrigant to prevent reinfection of the root canals and improve the outcomes of endodontic treatment. Nevertheless, animal and clinical studies should still be carried out to determine the efficacy of SDF in endodontics.

## Figures and Tables

**Table 1:** General characteristics and the outcomes of each study included in this review^∗^.

Study (authors and year)	Growth medium/samples (*n*)	Intervention groups (*n*)	*E. faecalis* culture and growth evaluation method (*s*)	Exposure time	Antibacterial outcomes
Hiraishi et al., [27]	Membranes	(1) 3.8% SDF (2) Ca(OH)_2_ (3) 5.25% NaOCl (positive control) (4) 0.9% NaCl (negative control)	CFU	15 min and 60 min for each group	SDF and NaOCl were significantly more effective in reducing *E. faecalis* than Ca(OH)_2_. No difference between SDF and NaOCl.
Mathew et al. [28]	Single rooted teeth (*n* = 44)	(1) 3.8% SDF (2) 2% CHX (3) 0.9% NaCl (4) 0.9% NaCl (no *E. faecalis*)	CFU	24 h	SDF and CHX had similar efficacy in reducing *E. faecalis*.
Al-Madi et al., [25]	Dentin discs (*n* = 70)	(1) 3.8% SDF (2) 2% CHX (3) 5.25% NaOCl (4) Saline	CLSM	10 min	NaOCl had the highest bactericidal effect. SDF had a higher bactericidal activity than CHX.
Abrar et al., [18]	Premolars (*n* = 40)	(1) PDT with MB (2) 5.25% NaOCl +17% EDTA (3) 3.8% SDF (4) 2% CHX	CLSM	(1) 180 s (2) 60s (3) 120 s (4) 120 s	All treatments had comparable efficacy against *E. faecalis*.
Minavi et al., [19]	Bovine dentin discs (*n* not stated)	(1) 38% SDF (2) 3.8% SDF (3) 0.38% SDF (4) 5.25% NaOCl (5) 2% CHX (6) PBS	Disc diffusion assays (for antimicrobial activities of 38% SDF, 3.8% SDF, and 0.38% SDF). SEM (for colonization levels after 3.8% SDF, NaOCl, 2% CHX, and PBS treatments)	2 min for all groups.	38% and 3.8% SDF had antibacterial effect and both were higher than 0.38% SDF. Substantivity of 3.8% SDF was comparable to CHX but greater than NaOCl.
Briseño-Marroquín et al., [26]	Schaedler bouillon and agar plates (*n* not stated)	(1) 38% SDF (2) 19% SDF (3) 9.5% SDF (4) 3.8% SDF (5) 38% SDF+KI (6) 19% SDF+KI (7) 9.5% SDF+KI (8) 3.8% SDF+KI	CFU	30 s	All groups had similar antibacterial effect against *E. faecalis*. KI did not have any impact on the antibacterial efficacy of SDF.

^∗^SDF: silver diamine fluoride; KI: potassium iodide; NaOCl: sodium hypochlorite; CHX: chlorhexidine; AgNP: silver nanoparticles; Ca(OH)_2_: calcium hydroxide.

**Table 2:** A list of the items assessed to evaluate the overquality of the studies included in this review^∗^.

Section/topic	Item number	Checklist items
Title	1a	The title must identify the study as being laboratory-based, e.g. “laboratory investigation” or “*in vitro*,” or “*ex vivo*” or another appropriate term
	1b	The area/field of interest must be provided (briefly) in the title

Keywords	2a	At least two keywords related to the subject and content of the investigation must be provided

Abstract	3a	The rationale/justification of what the investigation contributes to the literature and/or addresses a gap in knowledge must be provided
	3b	The aim/objectives of the investigation must be provided
	3c	The body of the abstract must describe the materials and methods used in the investigation and include information on data management and statistical analysis
	3d	The body of the abstract must describe the most significant scientific results for all experimental and control groups
	3e	The main conclusion(s) of the study must be provided

Introduction	4a	A background summary of the scientific investigation with relevant information must be provided
	4b	The aim(s), purpose(s) or hypothesis(es) of an investigation must be provided ensuring they align with the methods and results

Materials and methods	5a	A clear ethics statement and the ethical approval granted by an ethics board, such as an institutional review board or institutional animal care and use committee, must be described
	5b	When harvesting cells and tissues for research, all the legal, ethical, and welfare rights of human subjects and animal donors must be respected and applicable procedures described
	5c	The use of reference samples must be included, as well as negative and positive control samples, and the adequacy of the sample size justified
	5d	Sufficient information about the methods/materials/supplies/samples/specimens/instruments used in the study must be provided to enable it to be replicated
	5e	The use of categories must be defined, reliable and be described in detail
	5f	The numbers of replicated identical samples must be described within each test group. The number of times each test was repeated must be described
	5 g	The details of all the sterilization, disinfection, and handling conditions must be provided, if relevant
	5 h	The process of randomization and allocation concealment, including who generated the random allocation sequence, who decided on which specimens to be included and who assigned specimens to the intervention must be provided (if applicable)
	5i	The process of blinding the operator who is conducting the experiment (if applicable) and the examiners when assessing the results must be provided
	5j	Information on data management and analysis including the statistical tests and software used must be provided

Results	6a	The estimated effect size and its precision for all the objective (primary and secondary) for each group including controls must be provided
	6b	Information on the loss of samples during experimentation and the reasons must be provided, if relevant
	6c	All the statistical results, including all comparisons between groups must be provided

Discussion	7a	The relevant literature and status of the hypothesis must be described
	7b	The true significance of the investigation must be described
	7c	The strength(s) of the study must be described
	7d	The limitations of the study must be described
	7e	The implications for future research must be described

Conclusion(s)	8a	The rationale for the conclusion(s) must be provided
	8b	Explicit conclusion(s) must be provided, i.e. the main “take-away” lessons

Funding and support	9a	Sources of funding and other support (such as supply of drugs, equipment) as well as the role of funders must be acknowledged and described

Conflicts of interest	10a	An explicit statement on conflicts of interest must be provided

^∗^In accordance with a modified version of the Preferred Reporting Items for Laboratory studies in Endodontology (PRILE) guidelines developed by Nagendrababu et al., [22].

**Table 3:** Results of the quality assessment of the studies included in this review^∗^.

Section/topic	Item number	Hiraishi et al., [27]	Mathew et al., [28]	Al-Madi et al., [25]	Abrar et al., [18]	Minavi et al., [19]	Briseño-Marroquín et al., [26]
Title	1a	0	1	0	0	0	0
	1b	1	1	1	1	1	1

Keywords	2a	1	1	1	1	1	1

Abstract	3a	0	1	1	0	1	0
	3b	1	1	1	1	1	1
	3c	0.5 (no statistics details)	0.5 (no statistics details)	1	1	0.5 (no statistics details)	0.5 (no statistics details)
	3d	1	1	1	1	1	1
	3e	1	1	1	1	1	1

Introduction	4a	1	1	1	1	1	1
	4b	1	1	1	1	1	0

Materials and methods	5a	0	0	0	1	0	1
	5b	0	0	0	0	0	1
	5c	1	0.5 (+/- control not defined)	1	0.5 (+/- control not defined)	1	0.5 (+/- control not defined)
	5d	1	1	1	1	1	1
	5e	1	1	1	1	1	1
	5f	0	0	1	1	1	0
	5g	0	0	1	0	0	0
	5h	0	1	0	1	0	0
	5i	0	0	0	0	0	0
	5j	0.5 (no software)	0	0.5 (no software)	0.5 (no software)	1	1

Results	6a	0	0	0	0	0	0
	6b	0	0	0	0	0	0
	6c	1	0	1	1	1	1

Discussion	7a	1	0.5 (status of hypothesis not described)	0.5 (status of hypothesis not described)	1	0.5 (status of hypothesis not described)	0.5 (status of hypothesis not described)
	7b	1	1	1	1	1	1
	7c	0	0	0	1	1	0
	7d	0	0	0	1	1	0
	7e	1	0	1	1	1	0

Conclusion(s)	8a	1	1	0	1	0	0
	8b	1	1	1	1	1	1

Funding and support	9a	0	0	1	1	0	1

Conflicts of interest	10a	0	0	1	0	1	1

Total score (out of 32)		17	15.5	21	23	21.5	17.5

^∗^Descriptions of the item numbers are provided in Table 2.
